# Physical Activity and Quality of Life in Severely Obese Adults during a Two-Year Lifestyle Intervention Programme

**DOI:** 10.1155/2015/314194

**Published:** 2015-01-13

**Authors:** Randi Jepsen, Eivind Aadland, Lesley Robertson, Ronette L. Kolotkin, John Roger Andersen, Gerd Karin Natvig

**Affiliations:** ^1^Faculty of Health Studies, Sogn og Fjordane University College, P.O. Box 523, 6803 Førde, Norway; ^2^Department of Global Public Health and Primary Care, University of Bergen, 5020 Bergen, Norway; ^3^Red Cross Haugland Rehabilitation Centre, 6968 Flekke, Norway; ^4^Quality of Life Consulting, Durham, NC 27705, USA; ^5^Department of Community and Family Medicine, Duke University School of Medicine, Durham, NC 27708, USA; ^6^Department of Surgery, Førde Central Hospital, 6807 Førde, Norway; ^7^Morbid Obesity Centre, Vestfold Hospital Trust, 3103 Tønsberg, Norway

## Abstract

It is unknown how changes in physical activity may affect changes in quality of life (QoL) outcomes during lifestyle interventions for severely obese adults. The purpose of this study was to examine associations in the patterns of change between objectively assessed physical activity as the independent variable and physical, mental, and obesity-specific QoL and life satisfaction as the dependent variables during a two-year lifestyle intervention. Forty-nine severely obese adults (37 women; 43.6 ± 9.4 years; body mass index 42.1 ± 6.0 kg/m^2^) participated in the study. Assessments were conducted four times using Medical Outcomes Study Short-Form 36 Health Survey (SF-36), Obesity-Related Problems (OP) scale, a single item on life satisfaction, and accelerometers. The physical component summary (PCS) score and the mental component summary (MCS) score were used as SF-36 outcomes. Associations were determined using linear regression analyses and reported as standardized coefficients (stand. coeff.). Change in physical activity was independently associated with change in PCS (stand. coeff. = 0.35, *P* = .033), MCS (stand. coeff. = 0.51, *P* = .001), OP (stand. coeff. = −0.31,  *P* = .018), and life satisfaction (stand. coeff. = 0.39, *P* = .004) after adjustment for gender, age, and change in body mass index.

## 1. Introduction

Severely obese adults seeking lifestyle interventions report impaired physical, mental, and obesity-specific quality of life (QoL) [1–3]. Thus, several studies have included QoL as a primary outcome in evaluation of multicomponent lifestyle interventions for these individuals [4–7]. These studies have proposed physical activity to be a contributor to unexplained improvements in QoL [4–7]. Danielsen et al. [4] and Karlsen et al. [5] demonstrated improvements in the physical component summary (PCS) score and mental component summary (MCS) score of the Medical Outcomes Study Short-Form 36 Health Survey (SF-36) at the end of one-year, partly residential interventions. Blissmer et al. [7] reported similar findings after a six-month, outpatient intervention for overweight to obese adults. Repeated measures in two of these studies revealed improvements in both PCS and MCS after an initial, intensive intervention phase whereas longer-term maintenance varied [4, 7]. The results reported by Blissmer et al. [7] were independent of weight loss while Danielsen el al. [4] revealed positive associations between weight loss and improvements in PCS but not MCS. With regard to obesity-specific QoL, the Swedish Obese Subjects (SOS) study developed and used the instrument Obesity-Related Problems (OP) scale. After four [2] and ten years [8], the authors found significant improvements in severely obese adults who had received “conventional treatment.” However, the treatment was not standardised but was provided in accordance with local routines by many primary health care centres. Improvements in OP were positively correlated with weight loss [2, 8]. In contrast, despite weight regain, Kaukua et al. [6] demonstrated improvements in OP at the end of a two-year follow-up of severely obese individuals who completed a four-month, outpatient intervention.

Compared to physical, mental, and obesity-specific QoL, life satisfaction is a broader QoL construct representing a subjective and global assessment of all major dimensions of life [9]. Using single item measures on life satisfaction, obesity was associated with impaired life satisfaction in two U.S. population studies [10, 11], whereas a Danish epidemiological study, controlling for a cluster of lifestyle-related factors including body mass index (BMI), found independent positive associations between self-reported physical activity and life satisfaction [12].

Multicomponent lifestyle interventions for severe obesity aim for a sustainable change of behaviour related to diet and physical activity [13]. Physical activity is beneficial for body composition and fitness in obese individuals undergoing dietary energy restriction [14] and reduces adverse cardiovascular outcomes of obesity [15, 16]. In the eight-year follow-up of the Look AHEAD study, self-reported physical activity was associated with initial and maintained weight loss in overweight to obese subjects with type 2 diabetes [17]. Cross-sectional, unadjusted analyses in studies on treatment-seeking severely obese adults have demonstrated positive correlations between self-reported physical activity and PCS [18, 19] and MCS [18]. We found positive independent associations between objectively assessed physical activity and life satisfaction prior to a lifestyle intervention for severely obese adults [20] and did a series of studies on patterns of change in the participants during the intervention. We used accelerometers to objectively measure physical activity, collected data at multiple time points, and found positive associations in the patterns of change between physical activity and aerobic fitness [21], fat mass [22], and lipoproteins [23], confirming the importance of physical activity for clinical and anthropometric outcomes in lifestyle interventions. To our knowledge, a similar design has not been used to examine associations between change in physical activity and QoL outcomes over time. Therefore, the present study examines associations between change in objectively assessed physical activity as the independent variable and change in physical, mental, and obesity-specific QoL and life satisfaction as the dependent variables during a two-year, multicomponent lifestyle intervention.

## 2. Design and Methods

This study is part of the Haugland Obesity Study, a prospective cohort study on severely obese adults who participated in a publicly funded two-year lifestyle intervention at Red Cross Haugland Rehabilitation Centre (RCHRC) in Western Norway. Data were collected between February 2010 and October 2012 and the present study used data from four time points: baseline prior to the intervention (*T*0), six weeks later, at the end of the first residential stay, (*T*1), and prior to the residential stays one (*T*2) and two (*T*3) years from baseline.

Referral of patients was done by general practitioners in accordance with the right to admission to the Norwegian specialist health services (i.e., BMI ≥ 40 kg/m^2^ or ≥ 35 kg/m^2^ with comorbidities) [24]. In total, 53 eligible patients from the age of 18 to 60 years, divided into four groups, had their first residential stay between February 2010 and October 2011. Exclusion criteria included previous obesity surgery or referral to obesity surgery; severe cardiovascular disease; pregnancy; substance or alcohol abuse; and impaired physical functioning or mental problems which could interfere with adherence to the intervention.

### 2.1. Intervention

The intervention has been described in detail previously [25]. Briefly, the patients spent a total of 15 weeks at RCHRC divided into four stays of six, three (after three months), three (at year one), and three (at year two) weeks. A multiprofessional team managed the intervention. The overall goal was to improve the QoL of the patients, while weight loss, improved mental health and physical fitness, and reduction of obesity-related medical problems served as secondary goals. The group-based cognitive behavioural therapy, consisting of eleven sessions over two years, targeted QoL and self-management of physical activity and eating [26]. Scheduled physical activity in the residential periods consisted of brisk walking, swimming, strength training, ball games, and aerobics and amounted to nine to eleven hours weekly divided into bouts of 20–60 minutes. Each patient developed a plan for physical activity, modified to his or her preferences, limitations, and home situation. The diet followed the Nordic Nutrition Recommendations [27] and consisted of three high-fibre, low-fat, and energy-reduced meals and two to three snacks. The patients were advised to follow a similar diet at home. In the home periods, patients kept physical activity diaries which they sent to RCHRC on a monthly basis. There was no other follow-up between the residential periods.

### 2.2. Quality of Life Measures

#### 2.2.1. SF-36, Version 1.2

This is a 36-item measure of general health-related QoL. PCS and MCS are computed from the eight SF-36 subscales. PCS ranges from 15.4 to 62.1 and MCS from 10.1 to 64.0 (with higher scores representing better QoL) [28, 29]. The PCS and MCS have been standardised to a population normal distribution, with a mean of 50 and a standard deviation (SD) of 10. SF-36 has been widely applied in obesity research [1, 30], discriminates between subgroups of severely obese adults [1], and is sensitive to change during lifestyle interventions [4, 5].

#### 2.2.2. OP Scale, Version 1.2

This is an eight-item measure of obesity-specific QoL including questions about restaurant visits, holidaying, participation in community activities, swimming in public places, trying on and buying clothes, and intimate/sexual situations. The calibrated score ranges from 0 to 100 (<40 mild, ≥40 to <60 moderate, ≥60 to <80 severe, and ≥80 extreme problems) [2]. OP is reliable and valid in severely obese adults [2, 31]. In the present study, the internal consistency at baseline was excellent with a Cronbach alpha coefficient of 0.91.

#### 2.2.3. Life Satisfaction

Life satisfaction was assessed using a global question on current satisfaction with life with seven response alternatives from “very satisfied” to “very dissatisfied.” One-item measures on life satisfaction have demonstrated reliability [32] and validity in health research [33, 34].

### 2.3. Physical Activity

To assess physical activity, we used the ActiGraph GTI M accelerometer (ActiGraph, Pensacola, FL, USA), which is an electronic movement sensor. The accelerometer registers vertical acceleration and converts it into the unit “counts” which increase with the magnitude of the work rate for walking. The participants were instructed to wear the accelerometer over the right hip for seven consecutive days while awake, except during water activities. The *T*0, *T*2, and *T*3 assessments took place during home periods, while the *T*1 assessment was carried out at the end of the first residential period. The ActiGraph software ActiLife v. 5.3 was used for the data analysis. The criterion for a valid measure was wear-time of ≥ ten hours per day for ≥ four days. Non-wear-time was defined as periods of ≥60 consecutive minutes without counts, allowing for up to two minutes of counts within these 60 minutes [35, 36]. The overall physical activity, given as counts per minute, was calculated as total counts divided by total valid wear-time. The accelerometer has shown validity in severely obese individuals [37] and accelerometer assessed physical activity offers more accuracy than self-reported data [38].

### 2.4. Sociodemographic Information and Anthropometry

Sociodemographic information was self-reported on questionnaires. Health professionals collected the anthropometric data. Height was measured in the standing position without shoes using a stadiometer and reported to the nearest 0.5 cm. Fat mass and weight were measured on a bioelectrical impedance analysis device (BC 420S MA, Tanita Corp., Tokyo, Japan) in the morning, in a fasting state, in light clothes, and after voiding. Weight was reported to the nearest 0.1 kg. Waist circumference was measured twice at exhalation at the level of the umbilicus and reported as the mean of the two measurements.

### 2.5. Ethics

We obtained written, informed consent from all participants prior to the study in accordance with the Helsinki Declaration. Ethical approval was given by the Regional Committee for Medical and Health Research Ethics for South-East Norway (registration number 2010/159).

### 2.6. Statistical Analysis

The scores on life satisfaction were reversed before analyses so that higher scores indicated better satisfaction with life. BMI was calculated as weight in kilograms divided by height in meters squared. Subject characteristics are presented as means and SD for continuous variables and percentages for categorical data. Observed values for QoL measures are presented as means and SD. The effect size (ES) for differences between PCS and MSC population norms [39] and the study population scores at the four time points were calculated by subtracting the norms from the mean score of the participants divided by the SD of the latter. We performed an attrition analysis using the chi-squared test for difference in gender and the independent samples *t*-test for differences in other variables.

A linear mixed model based on restricted maximum likelihood estimation with random intercept for subjects was used in all analyses for change (Δ) over time [40], using least significant difference from baseline. The associations between the independent and the dependent variables were analysed using linear regression, applying delta scores between each time point (Δ*y*_1_ = *y*_1_ − *y*_0_; Δ*x*_1_ = *x*_1_ − *x*_0_; Δ*y*_2_ = *y*_2_ − *y*_1_, etc.) [40], giving a total of *n* = 73 (PCS and MCS), 72 (OP), and 71 (life satisfaction) observations. For physical activity, PCS, MCS, OP, life satisfaction, and BMI, the differences between *T*0 and *T*1 (Δ1), *T*1 and *T*2 (Δ2), and *T*2 and *T*3 (Δ3) were used. Gender, age, and change in BMI served as covariates in the adjusted regression analyses. A 1000-repetition bootstrap analysis was used to calculate 95% confidence intervals (CI) of the regression coefficients.

The changes from baseline to each of the time points in QoL measures, physical activity, and BMI, obtained from the linear mixed model, are presented as means with 95% CI. The ES for changes in the dependent variables were calculated by subtracting the mean *T*1, *T*2, and *T*3 estimates from the mean *T*0 estimate, divided by the SD of *T*0. Weight loss was calculated as percent change from baseline. A secondary analysis was performed using a baseline-observation-carried-forward approach for missing values.

Effect sizes were judged against the standard criteria proposed by Cohen: trivial (<0.2), small (0.2 to <0.5), moderate (0.5 to <0.8), and large (≥0.8) [41].

Calculation of sample size and power was done using the GPower version 3.1. Statistical analyses were conducted using SPSS for Windows, version 20.0 (SPSS Inc., Chicago, USA). A two-sided *P* value of ≤ 0.05 indicated statistical significance.

## 3. Results

**Figure 1:**
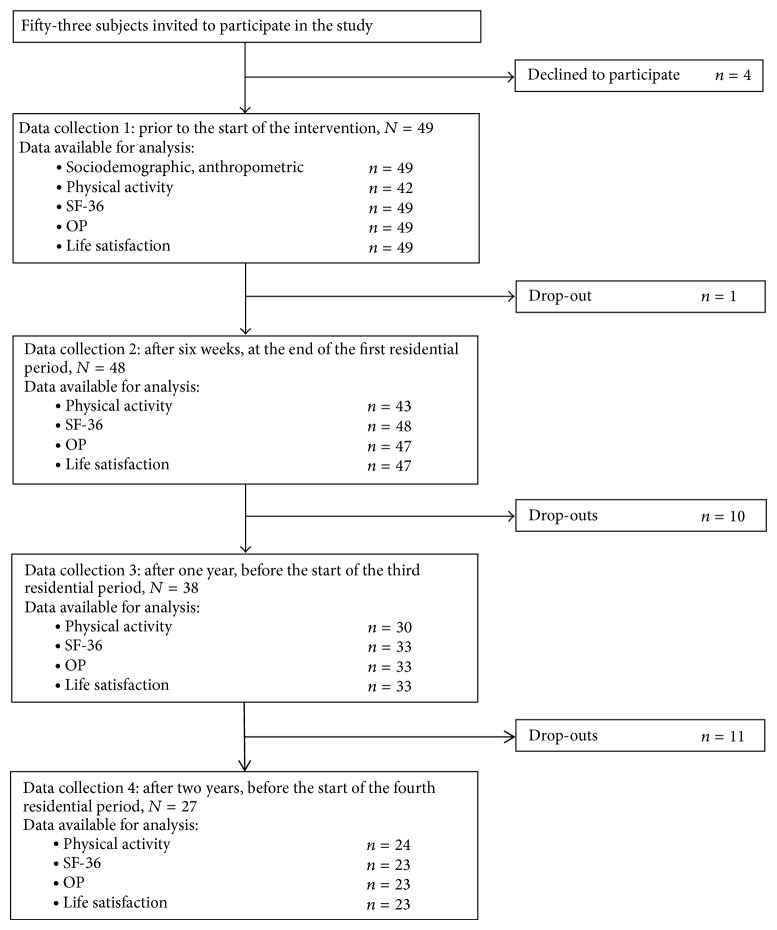
Flow chart for the prospective study of severely obese adults in a two-year lifestyle intervention, Medical Outcomes Study Short-Form 36 Health Survey: SF-36; Obesity-Related Problems (OP) scale.

Forty-nine patients (37 women; 43.6 ± 9.4 years; BMI 42.1 ± 6.0 kg/m^2^) consented to participate in the study. Baseline sociodemographic and anthropometric characteristics are presented in Table 1. At year two, 16 women and six men (44.9%) were lost to follow-up (Figure 1). Five withdrew from the study due to problems with the study protocol. The rest dropped out of the intervention itself, due to referral to obesity surgery, pregnancy, reaching personal weight goals, health problems, inability to attend the residential stays, or unknown reasons. The number of participants at each time point is noted in Table 2. The noncompleters did not differ from the completers with regard to gender, age, BMI, physical activity, QoL measures, or changes from *T*0 to *T*1 in BMI, physical activity, or QoL measures.

Table 2 documents changes in the QoL measures and the related ES. Over the first six weeks, all scores improved significantly. After two years, MCS and life satisfaction had returned to baseline levels, whereas the improvement in PCS was partly maintained. OP showed a different pattern with continuous improvements. The ES for within-group change was small for OP, moderate for PCS and MCS, and large for life satisfaction after six weeks. At year one, the ES was small for OP and moderate for PCS. Finally, at year two, the ES were small for PCS and moderate for OP [41]. Physical activity increased significantly during the first residential stay and was partly maintained at year one. At year two, it had returned to baseline level. Weight loss peaked at year one with 6.4% (Table 3).

Scores on PCS, MCS, OP, and life satisfaction are presented in Table 4 including ES for differences between the study population and the Norwegian SF-36 population norm [39]. For PCS, the difference was small at baseline and trivial at year two [41]. For MCS, it was trivial both at baseline and after two years. At baseline, the participants reported moderate obesity-related problems. Thereafter, the scores on OP reduced to mild problems [2].

**Figure 2:**
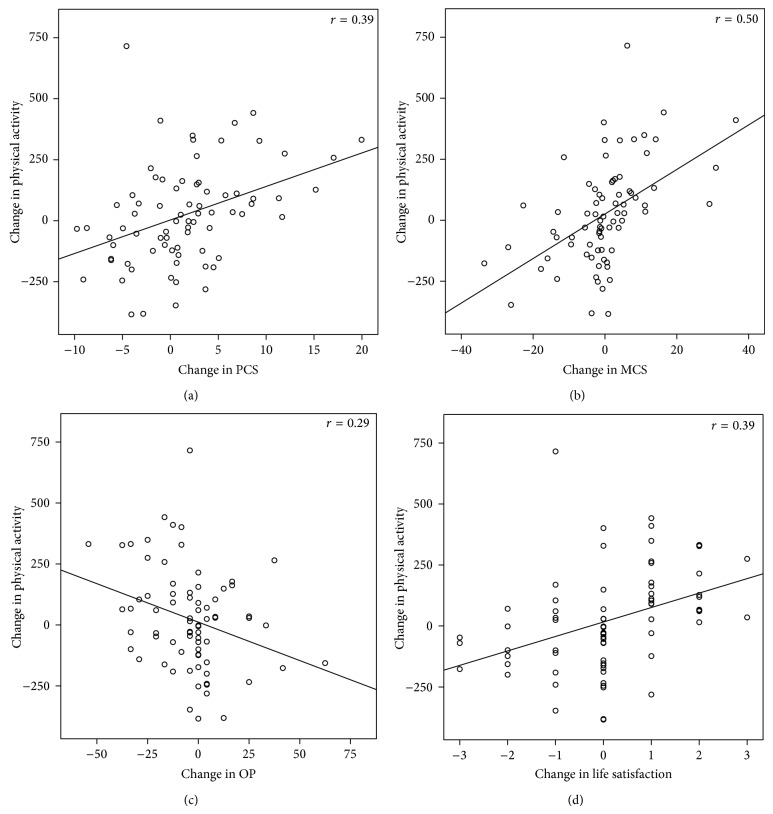
(a)–(d) Correlations between change in accelerometer assessing physical activity (counts per minute) and change in (a) PCS, (b) MCS, (c) OP, and (d) life satisfaction during the two-year lifestyle intervention for severely obese adults. PCS (physical component summary) score of the Medical Outcomes Study Short-Form 36 Health Survey (SF-36). Scale: 15.4–62.1. Higher scores represent better health-related quality of life. MCS (mental component summary) score of the Medical Outcomes Study Short-Form 36 Health Survey (SF-36). Scale: 10.1–64.0. Higher scores represent better health-related quality of life. OP: Obesity-Related Problems scale. Scale: 0–100. Higher scores represent more obesity-related problems. Life satisfaction. Scale: 1–7. Higher scores represent better satisfaction with life.

Figure 2 illustrates that correlations between change in physical activity and change in QoL measures were strongest for MCS and weakest for OP. This is also demonstrated in Table 5 which presents the results of the regression analyses. In the adjusted analyses, changes in PCS, MCS, OP, and life satisfaction were significantly associated with change in physical activity. The explained variance was moderate for PCS and MCS and small for OP and life satisfaction [41]. Change in BMI was correlated with change in PCS, MCS, and life satisfaction in the unadjusted analyses; however, this association was not statistically significant in the full models. Replacement of change in BMI with change in waist circumference or fat mass did not alter any results (data not shown). We tested for the interaction between physical activity and gender in all four models and found that the women had a stronger association between change in physical activity and change in PCS than the men (*P* = 0.012).

This study has 71 to 73 observations for the main outcomes (Table 5). Given 71 observations, a power of 0.80, and significance level of 0.05, the study should have power to detect a standardized coefficient of 0.32 (medium effect size).

## 4. Discussion

Results from this study suggest that change in physical activity is independently associated with change in physical, mental, and obesity-specific QoL and life satisfaction in severely obese adults participating in a lifestyle intervention. In correspondence with the present study, an independent dose-response relationship between exercise and physical and mental SF-36 subscales was reported from a randomized, controlled pre-post study on a six-month intervention for overweight to obese, menopausal, and hypertensive women [42]. A cross-sectional study on overweight to obese subjects with type 2 diabetes also reported associations between self-reported physical activity and MCS, but not PCS, independent of BMI [43]. By contrast, Ross et al. [44] reported that physical fitness, which may be a proxy for physical activity, did not mediate the association between weight reduction and improvements in SF-36 subscales in obese women enrolled in a six-month intervention. However, the six-minute walk test, which was utilized to assess fitness in the study by Ross et al. [44], may lack accuracy in pre-post design in obesity research [21]. Bond et al. [19] used self-reported data on physical activity from two time points and found associations with PCS, but not MCS, in obesity surgery-seekers, although these results did not control for BMI. With respect to obesity-specific QoL, Kaukua et al. [6] described improvements in OP alongside fluctuations in self-reported physical activity during a lifestyle intervention but did not examine correlations. Regarding life satisfaction, our cross-sectional baseline study from the Haugland Obesity Study found an independent association with physical activity [20]. Population data revealed a positive relationship with self-reported physical activity, though this relationship did not control for BMI [10]. A comparison of lifestyle interventions found no difference in life satisfaction between intervention and control groups during a one-year follow-up [35]. However, physical activity was not included in the analyses. So some studies do not support our finding of associations between change in physical activity and change in all QoL outcomes. One explanation may be that the intervention of the present study clearly differed from other lifestyle interventions in that the overall goal was improvement of QoL. Other interventions have weight management [2, 7] or behaviour change related to physical activity and diet (which should lead to weight loss) [4–6] as primary goals. The inconsistencies across studies may also relate to the cross-sectional design of several of them [10, 20, 43], problems with the reliability of self-reported physical activity [36], the variety of weight classes included in the studies, and other heterogeneities of participants, context, or research designs.

Interestingly, although the unadjusted analyses revealed correlations between changes in BMI and PCS, MCS, and life satisfaction, weight loss did not moderate the associations between the independent and the dependent variables in the adjusted analyses. Associations between weight loss and improvements in PCS have been found in several studies on patients undergoing lifestyle interventions [4, 5, 37] but not in the study by Blissmer et al. [7]. Findings on the relationship between weight loss and MCS are also inconsistent. Neither Danielsen et al. [4] nor Pazzagli et al. [37] found this association in lifestyle interventions. Neither did Kolotkin et al. [38] after obesity surgery, whereas Karlsen et al. [5] did in a pooled sample of obesity surgery patients and lifestyle intervention completers. Our finding on OP, as the only variable which had a nonsignificant correlation with change in BMI in the unadjusted analyses, is contradictive to the SOS study which demonstrated short- and long-term decreases in obesity-related problems associated with weight loss [2, 8]. Other obesity-specific measures have also shown associations with weight loss [38]. More research is needed to fully understand the relationship between weight loss and QoL. Noticeably, with regard to the purpose of the present study, none of the above-mentioned studies included physical activity as a variable.

The positive boost in all QoL measures over the first residential stay is noteworthy. PCS and MCS even increased above population norms [39]. Similar improvements on PCS and MCS have been found by Danielsen et al. [4] after an initial, in-patient period and Kaukua et al. [6] at the end of a four-month outpatient programme using the RAND-36 questionnaire, equivalent to the SF-36 [45], and the OP. However, despite the statistically significant effect of physical activity on QoL measures in our analyses, many of the improvements in the dependent variables were left unexplained. Thus, several other aspects may have played a role, such as experience of peer support [46, 47], reduction of anxiety [4], improved eating pattern [48], and improved self-regulation and self-efficacy [49] which have been demonstrated by others after intensive intervention phases, as well as our explicit intervention focus on improvement of QoL. Regarding the patterns of longer-term changes, there are variations across studies. Our finding that MCS had returned to baseline at year two while moderate ES for the change in PCS was maintained is opposite to two-year changes reported by Blissmer el al. [7] on overweight to obese subjects. The ES for one-year changes reported for lifestyle intervention completers by Karlsen et al. [5] is similar to the ES of the present study for PCS (=0.47), but higher (=0.32) for MCS. Yet the baseline scores on PCS (mean = 39, SD = 10) and MCS (mean = 42, SD = 11) were lower compared to our study which may indicate a greater potential for long-term improvements. So, although lifestyle treatment-seekers generally report better QoL than obesity surgery-seekers and worse than non-treatment-seekers [1, 3], variations across study populations in lifestyle interventions may contribute to disparities in research outcomes. Moreover, it may be unrealistic to expect the initial peaks in PCS, MCS, and life satisfaction to last in the long run. In fact, that would imply better PCS and MCS scores than in the general population [39]. For future studies, examining how favourable outcomes of intensive phases of lifestyle interventions can be maintained, at least partly, over time will be worthwhile. And, in that regard, our finding of continued improvement in the OP is of interest. An intervention that can help participants experience less obesity-related problems, despite modest weight loss, may be seen as positive.

Change of health-related behaviours is challenging [13] and the interaction of time is an aspect that deserves consideration. Often, lifestyle interventions are reported to be shorter than the present, for example, four months [6], six months [7], or one year [4, 5]. The SOS study has published data on long-term follow-up, but the length of the included conventional treatment is not standardised or described [2, 8]. The Look AHEAD study, though including patients of all weight classes from overweight to severely obese, does provide detailed information about lifestyle intervention for adults with type 2 diabetes and is unique with regard to the large number of participants (*N* = 5,145), the randomisation, and the length of the intervention, that is, eight years [17]. Clearly, more studies are needed to develop a better understanding of long-term effects of lifestyle interventions on QoL outcomes.

### 4.1. Methodological Considerations

The present study has several strengths. First, the use of accelerometers to collect data on physical activity increased reliability over self-reporting [36]. Second, as recommended, both general and condition-specific QoL instruments were used [2]. Third, data was collected at four time points and used to reveal associations of patterns of change. Since change of health-related behaviour is complex, increase and maintenance of physical activity are challenging, and subjective constructs like QoL are not straightforward, these findings on patterns of change contribute uniquely to the body of knowledge about lifestyle interventions for severely obese adults.

A limitation of this study was that the number of participants lost to follow-up challenged the statistical power. High attrition is not unusual in research on lifestyle interventions [6, 7] but differences between completers and noncompleters vary across studies from none [5, 6] to one [4, 7] or some [50]. In the present study, the attrition analyses revealed no statistical difference in key variables between the drop-outs and the completers. The secondary analysis confirmed the statistical level of change in the QoL measures, counts per minute, and BMI (data not shown). To deal with the challenge of statistical power, all valid data were included in the linear mixed model. Regarding the sample size, the study was powered to detect medium sized effect sizes as found in the regression analysis of this study. Inclusion of a control group amongst the referred patients was not possible due to their right to treatment [24]. This study examined associations and, therefore, causal relationships could not be inferred. And the study did not control for change of diet, a possible confounder in the associations we examined [51, 52]. However, physical activity has been found to contribute more to QoL than dieting [53]. In addition, we controlled for change in BMI which may be a proxy for diet, because generally diet modifications produce more weight loss than physical activity [54, 55]. Lastly, the patients were a self-selected, treatment-seeking group although public funding of the intervention gave equal access to all and, therefore, diminished the risk of socioeconomic bias [56].

## 5. Conclusions

It has been proposed that lifestyle interventions for obese individuals should focus less on weight loss as the primary outcome and pay more attention to independent benefits of physical activity such as reduction of obesity-related health hazards [57] and improvements of QoL [42]. The present study contributes uniquely to the literature on severe obesity, physical activity, and self-reported outcomes and indicates that improved QoL may be a valid result of increased physical activity in multicomponent lifestyle interventions. These findings should be further tested in various settings, in larger samples, and with control groups.

## Figures and Tables

**Table 1:** Characteristics of the adults with severe obesity at baseline, *N* = 49.

Age, mean (SD)	43.6 (9.4)
Gender, *n* (%)	
Women	37 (75.5)
Sociodemographic status, *n* (%)	
Married/cohabiting	31 (63.3)
Having children	27 (55.1)
College/university education	22 (44.9)
Employed	41 (83.7)
Anthropometrics, mean (SD)	
Body mass index, kg/m^2^	42.1 (6.0)
Weight, kg	123.9 (18.6)
Waist circumference, cm	128.3 (13.0)
Fat mass, %	58.2 (11.7)

Standard deviation: SD.

**Table 2:** Mixed-effect model estimates: mean changes (95% CI) in quality of life outcomes during the two-year lifestyle intervention for severely obese adults.

Measure	*T*1: change from *T*0	*T*2: change from *T*0	*T*3: change from *T*0
SF-36 physical component summary^a^	5.7 (7.4, 4.0) *P* < .001	4.4 (6.3, 2.5) *P* < .001	3.3 (5.5, 1.1) *P* = .004
Effect size	0.61	0.54	0.48
SF-36 mental component summary^b^	5.9 (8.6, 3.1) *P* < .001	−0.4 (−2.7, 3.5) *P* = .794	−1.8 (−1.8, 5.3) *P* = .327
Effect size	0.55	0.02	−0.06
Obesity-related problems scale^c^	−8.4 (−2.9, −13.9) *P* = .003	−11.2 (−5.0, −17.4) *P* = .001	−13.3 (−6.2, −20.4) *P* < .001
Effect size	0.30	0.49	0.57
Life satisfaction^d^	0.99 (1.30, 0.67) *P* < .001	0.47 (0.82, 0.12) *P* = .009	0.20 (0.60, 0.20) *P* = .324
Effect size	1.00	0.55	0.22

*T*0: before the intervention (*n* = 49); *T*1: after six weeks (*n* = 48); *T*2: year one (*n* = 38); *T*3: year two (*n* = 27).

^a^Scale 15.4–62.1: higher scores represent better quality of life, Medical Outcomes Study Short-Form 36 Health Survey.

^b^Scale 10.1–64.0: higher scores represent better quality of life, Medical Outcomes Study Short-Form 36 Health Survey.

^c^Scale 0–100: higher scores represent more obesity-related problems.

^d^Scale 1–7: higher scores represent better life satisfaction.

Significant *P* values (≤.05) in bold.

Effect sizes for the within-group changes were calculated by subtracting the mean estimates of follow-ups from the mean estimates at baseline divided by the SD of the latter. They were judged against the standard criteria proposed by Cohen: trivial (<0.2), small (0.2 to <0.5), moderate (0.5 to <0.8), and large (≥0.8) [41].

Confidence interval: CI.

**Table 3:** Mixed-effect model estimates: physical activity and BMI during the two-year lifestyle intervention for severely obese adults.

	*T*0	*T*1	*P* ^*^	*T*2	*P* ^*^	*T*3	*P* ^*^
	Mean (95% CI)	Mean (95% CI)		Mean (95% CI)			
Accelerometer assessed physical activity, CPM^†^	*n* = 42	*n* = 43	**<.001**	*n* = 30	**.036**	*n* = 24	.606
	276 (241, 311)	452 (417, 486)		327 (286, 368)		290 (244, 335)	
BMI, kg/m^2^	*n* = 49	*n* = 48	**<.001**	*n* = 38	**<.001**	*n* = 27	**.001**
	42.1 (40.3, 43.8)	40.1 (38.4, 41.8)		39.4 (37.6, 41.1)		40.7 (38.9, 42.5)	
Weight loss from *T*0, per cent		4.8		6.4		3.3	

*T*0: before the intervention; *T*1: after six weeks; *T*2: year one; *T*3: year two.

Significant *P* values (≤.05) in bold.

^*^ *P* values for change from *T*0.

Body mass index: BMI; confidence interval: CI; counts per minute: CPM.

^†^Mean physical activity of American obese adults: 288 CPM [58]. Mean physical activity of Norwegian obese women: 276 CPM, and men: 290 CPM [59]. Mean physical activity of American normal weight adults: 344 CPM [58]. Mean physical activity of Norwegian normal weight women: 352 CPM, and men: 368 CPM [59].

**Table 4:** Mean and standard deviation of quality of life outcomes during the two-year lifestyle intervention for severely obese adults.

	*T*0	*T*1	*T*2	*T*3	Population norm
SF-36^a^	*n* = 49	*n* = 47	*n* = 33	*n* = 23	
Physical component summary	45.3 (9.6)	51.2 (7.3)	50.5 (7.8)	49.9 (7.6)	49.0
Effect size	−0.39	0.30	0.19	0.12	
Mental component summary	48.4 (10.2)	54.0 (7.7)	48.6 (12.8)	47.8 (10.7)	49.0
Effect size	−0.06	0.65	−0.03	−0.11	
Obesity-related problems^b^	*n* = 49	*n* = 46	*n* = 33	*n* = 23	n/a
	44.6 (26.3)	36.7 (25.0)	31.8 (28.0)	29.7 (24.4)	
Life satisfaction^c^	*n* = 49	*n* = 46	*n* = 33	*n* = 23	n/a
	4.6 (0.9)	5.5 (1.0)	5.1 (0.9)	4.8 (0.9)	

*T*0: before the intervention; *T*1: after six weeks; *T*2: year one; *T*3: year two.

^a^Scale 0–100: higher scores represent better quality of life, Medical Outcomes Study Short-Form 36 Health Survey (SF 36). The SF-36 data for the norm population (*n* = 2,323) are adjusted for gender and age [39] and presented as means. Effect sizes for differences between the study participants and the norm population were calculated by subtracting the mean score of the population norm from the mean score of the study participants divided by the SD of the latter. They were judged against the standard criteria proposed by Cohen: trivial (<0.2), small (0.2 to <0.5), moderate (0.5 to <0.8), and large (≥0.8) [41].

^b^Scale 0–100: higher scores represent more obesity-related problems, Obesity-Related Problems scale.

^c^Scale 1–7: higher scores represent better life satisfaction.

**Table 5:** Reg. coeff. with 95% CI and stand. coeff. (*β*) for simple and multiple^a^ linear associations between change in physical activity as the independent variable and change in quality of life outcomes as the dependent variable.

	Change in PCS^b^			Change in MCS^b^			Change in OP^c^			Change in life satisfaction^d^		
	Reg. coeff. (95% CI)	*β*	*P*	Reg. coeff. (95% CI)	*β*	*P*	Reg. coeff. (95% CI)	*β*	*P*	Reg. coeff. (95% CI)	*β*	*P*
Gender: male												
Crude	0.31 (−2.66, 3.26)	.02	.838	2.10 (−2.63, 6.84)	.09	.380	−0.68 (−9.28, 7.93)	−.02	.876	−0.13 (−0.76, 0.51)	−.04	.691
Adj.	−0.65 (−3.39, 1.96)	−.05	.635	1.77 (−2.81, 6.73)	.07	.472	−1.40 (−11.89, 8.32)	−.03	.783	−0.22 (−0.97, 0.49)	−.07	.530
Age												
Crude	0.09 (−0.04, 0.23)	.13	.179	0.05 (−0.17, 0.27)	.04	.658	−0.18 (−0.58, 0.22)	−.09	.375	0.01 (−0.02, 0.04)	.06	.555
Adj.	0.04 (−0.13, 0.20)	.06	.650	0.08 (−0.18, 0.34)	.07	.537	−0.21 (−0.71, 0.24)	−.10	.363	0.003 (−0.027, 0.031)	.02	.852
Change of BMI												
Crude	−0.92 (−1.56, −0.29)	−.28	**.005**	−1.36 (−2.41, −0.32)	−.26	**.011**	1.82 (−0.09, 3.73)	.19	.061	−0.18 (−0.31, −0.04)	−.26	**.011**
Adj.	−0.34 (−1.32, 0.38)	−.11	.434	−0.03 (−1.44, 1.33)	−.01	.960	−0.24 (−3.03, 2.80)	−.03	.850	−.003 (−0.181, 0.142)	−.01	.974
Change of physical activity^e^												
Crude	0.011 (0.005, 0.017)	.39	**<.001**	0.028 (0.017, 0.039)	.50	**<.001**	−0.027 (−0.048, −0.007)	−.29	**.011**	0.003 (0.001, 0.004)	.39	**.001**
Adj.	0.010 (0.001, 0.019)	.35	**.033**	0.028 (0.014, 0.044)	.51	**.001**	−0.029 (−0.058, −0.008)	−.31	**.018**	0.003 (0.001, 0.004)	.39	**.004**
Adj. R^2^	0.13			0.22			0.04			0.11		

^a^All variables in the first column.

^b^Physical component summary (PCS) and mental component summary (MCS) of the Medical Outcomes Study Short-Form 36 Health Survey (SF-36). Continuous scales. Higher scores represent better quality of life.

^c^Obesity-Related Problems scale. Continuous scale. Higher scores represent more obesity-related problems.

^d^Continuous scale. Higher scores represent better life satisfaction.

^e^Accelerometer assessed.

Regression coefficients: reg. coeff.; confidence interval: CI; standardized coefficients: stand. coeff.; adjusted: adj.; body mass index: BMI.

Number of observations: change in PCS, 73; change in MCS, 73; change in OP, 72; change in life satisfaction, 71.

Significant *P* values (≤.05) in bold.
